# Effect of problem-based learning on students’ attitude towards learning physics: a cohort study

**DOI:** 10.12688/f1000research.125085.1

**Published:** 2022-11-01

**Authors:** Stella T. Kanyesigye, Jean Uwamahoro, Imelda Kemeza

**Affiliations:** 1 Education, National University of Rwanda, Kigali, Eastern, Rwanda; 2 Education, Mbarara University of Science and Technology, Mbarara, Uganda

**Keywords:** problem-based learning, teaching instruction, attitude, physics, expert-like attitude

## Abstract

**Background:** Attitude is a learning scale that informs which approach should be used to call students to school. It can be seen a supporting tool that informs teachers, policymakers, and researchers of the needs for raising interest in learning a certain subject, such as physics. This study aimed at determining the effect of problem-based learning on students’ attitude towards learning physics.

**Methods:** The study followed a quantitative approach with a quasi-experimental design employing cross-sectional survey techniques. The participants of the study were 419 13 ^th^-grade physics students of the 2020/2021 school year in both Ugandan government and private secondary schools. Among these students, one group was taught using problem-based learning instruction while another group was taught using traditional instruction for 12 weeks. Data were collected using a standardized tool called Views About Science Survey. Using Microsoft Excel 2016 and Statistical Package for Social Scientist version 23.0, descriptive and inferential statistics were used to determine a significant difference between experiment and control groups.

**Results:** It was found that both problem-based learning and traditional instructions caused a statistically significant positive effect on students’ attitudes towards physics. However, the experimental group gained more positive attitude than the control group as they were more inclined towards the expert-like attitude (thinking like a scientist in a domain) than their counterparts due to the problem-based learning approach they learned in.

**Conclusions:** Therefore, it was concluded that problem-based learning is a more effective method of teaching physics than traditional methods. Hence, we suggest that secondary school teachers need to adopt the use of problem-based learning in the teaching of science concepts, especially physics.

## Introduction

The world is presently faced with the challenge of getting graduates who possess the knowledge and skills required to solve difficult problems, gather and evaluate evidence, and interpret information received from various sources ( U.S. Department of Education Office of Innovation and Improvement, 2016). Learning and practicing science, technology, engineering, and mathematics (STEM) helps students gain these skills, which they use to understand the world around them and be curious in nature ( Andiema, 2016). STEM education enables students to gain knowledge and skills through solving problems from a multidisciplinary point of view and provides them with opportunities to obtain 21st-century skills to specialize in related fields ( Saraç, 2018) for their career endeavors. However, these graduates are reluctant to follow STEM subjects; others cannot deliver what is expected from their expertise due to the low interest they possess ( Gunel *et al.,* 2007; Ndihokubwayo *et al.,* 2021; Nyirahabimana, 2022).

We need to encourage our students to think scientifically or become expert physicists ( Madsen *et al.,* 2020). Scientific thinking is a type of information seeking that involves purposeful information seeking, including asking questions, testing hypotheses, making observations, recognizing patterns, and drawing conclusions. Various researchers ( Halloun & Hestenes, 1996b; Madsen *et al.,* 2020; Phillips & O’donnell, 2021; Stephens & Clement, 2010) involved in physics education research (PER) have undertaken studies of expert-like and novice-like problem-solving and attitude strategies. For instance, Phillips and O’donnell (2021) identified “expert approaches” as those possessed by physics PhDs and “novices” as introductory physics students. Thus, expert-like attitude is to perceive and think systematically and like a physicist. On other hand, folks-like attitudes are possessed by people with general and insufficient knowledge of physics. One of the earliest documenting instruments of such attitudes is the Views About Science Survey (VASS), which sought to distinguish between “expert” and “folk” views of physics ( Halloun, 1996; Phillips & O’donnell, 2021). The relative importance of the scientific thinking of experts and students is debatable because some teachers prefer to stress the importance of propositional thinking, while others believe that pictorial processes using the imagination are just as important as propositional knowledge ( Stephens & Clement, 2010).

Teacher-centered methods, commonly referred to as traditional or conventional instruction, allow the teacher to retain full control of the classroom and its activities ( Mpho, 2018) while students remain passive recipients of knowledge ( Karamustafaoglu, 2009). According to Hill (2002), an example of traditional instruction includes direct instruction/chalk and talk, which describes various whole-class expository teaching techniques. In this instruction, the teacher is an information provider, decides the content to be taught, does not motivate or encourage learners, and gives low facilitation; the learners, on the other hand, are lowly participating, have insignificant initiatives, are passive in the classroom interaction, and only respond to the teachers’ questions ( Nzeyimana & Ndihokubwayo, 2019). Teachers who employ this approach concentrate on the content of teaching and what they do in teaching by focusing on organizing, structuring, and presenting the course content in a way that is easier for the students to understand ( Sari *et al.,* 2006).

One of the most targeted practices in STEM education has been to improve the social aspects of learners, including attitude ( Nite *et al.,* 2015). Attitude is someone’s feeling, opinion, or behavior towards something. According to Arman (2018), attitude is a tendency to behave in a particular way; it is an internal state that influences students’ choices or decisions to act under certain conditions. Thus, in this study, we define attitude towards physics as the feeling, beliefs, and values possessed by students towards the subject conveyed in the form of like or dislike; and positive or negative reactions towards physics concepts. A positive attitude creates a positive identity, improves one’s health, creates possibilities, and makes one win friends; people with a positive attitude tend to be goal achievers, enjoy success, and seem to be happy by choice despite their circumstances; while people with negative attitude tend to drift through life complaining that nothing good ever happens; everything looks bad to them, and it becomes terrible ( Toler, 2016).

However, enrollment of science students into higher institutions has been low partly due to few students opting for science subjects at an advanced level of secondary education ( Nannyonjo *et al.,* 2009). Physics knowledge is thought to facilitate students in developing logical skills needed for problem-solving in various dimensions of life they encounter ( Eijkelhof & Kortland, 1998). However, students tend to have difficulty understanding physics concepts and solving related problems ( Kanyesigye *et al.,* 2022a; Sirait *et al.,* 2017). They look at physics as a challenging subject ( Ibrahim, 2019) and as a problematic one ( Ryan, 2013; Selçuk, 2010). They also consider the contents of physics to be mere facts and composed of formulas that need memorization, making them possess a negative attitude towards the subject ( Mbonyiryivuze, *et al.*, 2021).

Referring to previous research, according to Nite *et al.* (2015), students’ attitude was found to influence their performance significantly and consequently their decision to major in a STEM field. Olusola and Rotimi (2012) noted that students who have a negative attitude towards sciences, including physics, also tend not to like the subject teachers ( Olusola & Rotimi, 2012). Previous researchers such as Mbonyiryivuze *et al.* (2021); Andiema (2016), and Selçuk (2010) recommended the adoption of interactive pedagogical approaches in the teaching-learning process, including problem-based learning (PBL) instruction ( Kanyesigye & Kemeza, 2021) as a way of bringing about a desirable positive change in students’ attitude towards science and physics in particular.

Today’s education system focuses on training students to develop skills that enable them to work in a variety of situations ( Ndihokubwayo *et al.,* 2021). Like pragmatists, philosophers of education suggest that people learn by solving and learning from real problems they face in everyday life ( Richardson, 2003). Based on these philosophies, PBL was developed from the constructivism school of thought, where learners work themselves to generate new knowledge. PBL is defined as a method of inquiry where students solve difficulties, oddities, qualms, and problems in real life ( Dorimana *et al.,* 2021). PBL is one of the powerful teaching methods in reforming science education ( Allchin, 2013). The method acknowledges the importance of actively engaging students in their learning. It contextualizes the learning, which contributes both to student motivation and the making of meaning. According to Orozco and Yangco (2016), most of the students involved in PBL can share their opinions with others, use different approaches to analyze situations, and explore ways of solving problems. Therefore, the present study employs the extended constructivism theory of Albert Bandura’s social learning theory ( Bandura, 1985). This theory proposes that new behaviors can be acquired by observing and imitating others ( Bandura, 1985). Social learning theory fits our study because, through PBL, students interact with each other to reach a positive outcome. Students cooperatively share ideas ( Sibomana *et al.,* 2021) and then find the probable solution from a combined effort.

Students’ attitude towards learning can affect their success ( Sirait *et al.,* 2017). According to Ibrahim (2019), students’ negative attitude towards physics positively corresponds to low student achievement in the subject. Assessing students’ attitude is crucial for adapting to appropriate instruction. Madsen *et al.,* (2020) advised teachers and researchers that improving your students’ attitudes and beliefs about physics helps them more successfully learn physics content and helps develop their ability to think like a physicist. In this regard, research by Nteere *, et al.,* pointed out that the method of instruction employed by a teacher influences students’ attitude towards physics as a subject. However, research on how methods of instruction such as PBL effect the attitudes of students are limited. This study aimed to determine the effect of PBL on students’ attitudes towards physics. Two basic questions guided this study:

1. Does PBL instruction cause a statistically significant effect on students’ attitudes towards physics?
2. How does students’ attitude correlate with their performance scores?

## Methods

### Design of the study

This study aimed at determining the effect of PBL instruction on students’ attitude towards physics. The study followed a quantitative approach with a quasi-experimental design employing, specifically, a pre-test–post-test control group experimental design following a randomized solomon four group design ( Dimitrov & Rumrill, 2003) in which PBL as an intervention was applied on the experimental group while the control group was instructed using traditional instructions as elaborated in the literature review section.

### Sample and sampling methods

The study was conducted from January to April 2021 with 419 physics students of the 13 ^th^ grade (aged between 16 and 21) in Mitooma District-Southwestern Uganda. Selection of participating schools and allocation to the treatment and comparison groups were based on simple random sampling. Cluster sampling technique ( Creswell, 2014) was employed in this study where intact classes were used as units of analysis. 19 classes from 19 schools were employed. The number of students in each school can be found in our data article ( Kanyesigye *et al.,* 2022b). For instance, the sample of female students in the pre- and post-test groups was 53, while the sample of male students in the experimental group was 79. In the control group, there were 39 female students and 68 male students. Similarly, the sample of public-school students was 39 in the pre-test and post-test, while the sample of private school students was 92 in the experimental group. There were also 55 students in public school and 51 students in private school included in the control group. The population and sample sizes used in this study were taken at a 95% confidence interval in accordance with Morgan (2006). All secondary schools in Mitooma district are both day and boarding, with only two single girls’ schools. Thus, all schools included are day and boarding schools. Note that there was no exclusion criteria.

### Training of physics teachers in the PBL process

A two-day, six-hour professional training was organized on 10 ^th^ and 11 ^th^ January 2021 at Ruhinda Secondary School-Mitooma district and was attended by 30 physics teachers from Mitooma district in Southwestern Uganda. This training was designed as part of the study ( Kanyesigye *et al.,* 2022d). The participants were invited to the training depending on their teaching subject (physics) in Mitooma district. The main purpose of the professional training was to enhance secondary school in-service physics teachers’ knowledge of PBL and was guided by the following objectives:

1. To provide background information on the origin and importance of PBL
2. To provide skills on generating PBL questions
3. To provide skills on the presentation of a PBL lesson
4. To provide knowledge on the assessment of a PBL lesson

Participants were split into groups of five. The trainer for the Secondary Science and Mathematics (SESEMAT) program in the western region of Uganda and the first author served as facilitators for the formed groups. The SESEMAT trainer is one of the experts in Uganda that were first trained in the implementation of competence-based curriculum and appointed at the regional level by the government to train other teachers. As such, during the proposal development and development of the training content, this expert played a big role. So, inviting him to facilitate the training was based on his expertise and the fact that he had participated in the material development and validation of this study research instrument. The first author is a national examiner of physics. During proposal development, the author consulted the physics teachers (participants of this study) on which topics in physics generally posed greater challenges to students. Among other topics, the topic of waves was pointed out based on the generally poor performance of students in wave concepts and the fact that questions on wave concepts are also mostly dodged. So, the author took up the topic of waves for the PhD project. The roles for the participants and the training leader were defined at the start of the training. In formulating PBL questions, the topic of waves was selected as agreed prior to the course upon by majority of the participants, using direct messaging (WhatsApp) with the first author, in the training based on possession of prior knowledge. The aim of the training, was to provide the participants with the ability to draft real-life-based problems on the concepts of waves using online resources and textbooks.

### Implementation of the intervention

All activities were carried out throughout the study during normal class hours (four hours per week for four weeks). Before starting the experimentation, a pre-test was administered to both experimental and control groups in the first week — 10 physics teachers who handled the experimentation group first trained students about PBL strategies before starting the experiment. Experimental-1 (pre- and post-test design) accommodated 132 students, experimental-2 (post-test only design) accommodated 99 students, control-1 (pre- and post-test design) accommodated 107 students, and control-2 (post-test only design) accommodated 81 students. During the implementation, the teachers of the experimental groups in each lesson would purposively divide students into five to six-member groups, and each group would be presented a problem to research and after make a presentation to the whole class under the guidance of their respective teachers. This implementation was monitored at all levels by the researchers. Each group member, in one way or the other, had a responsibility to fulfill. The members were required to participate during the group discussions actively. Members in their groups were required to share their knowledge, ideas, and experiences about the solution of the given problem. Before group discussion, each member was expected to make individual study and be able to represent, communicate and evaluate and assess their learning either individually or at group level. In case of need for guidance, the teacher would pose open-ended general questions for students to think about critically. Students would, at the end of each activity, evaluate each other in relation to participation, preparation, interpersonal skills contribution to the progress of the group.

On the other hand, students in the control group were instructed (under supervision from the researchers) using traditional approaches where the teachers copied notes either from textbooks or from their notebooks and dictated them to students to write down in their notebooks. A few teachers would write about one or two problems on the chalkboard and again solve them as students watched. They would thereafter be referring the students to either textbooks and/past-papers for trial questions, and students’ solutions were hardly harmonized. The exercise lasted for twelve weeks, after which a post-test similar to the pre-test was administered to the participating students. Note that a researcher was present for all lessons across all groups. This was possible because different schools taught the topic of waves at different periods in the term. So, throughout the whole period of data collection, the authors were in the field. The notes from these observations can be found at Kanyesigye *et al.* (2022c).

### Data collection methods

**Figure 1.**
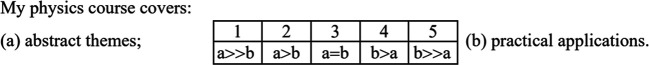
Example of Views About Science Survey type of item and its rating.

Data was collected using the Views About Science Survey (VASS) developed by Ibrahim Halloun ( Halloun, 1996; Halloun & Hestenes, 1996a, 1996b) focused on attitude. “Attitudes and beliefs surveys that are commonly used in physics courses (such as VASS) are about how students perceive the discipline of physics or their particular physics course” ( Madsen *et al.,* 2020, p.90). This tool is a valid and reliable tool to fit our research objectives and has two versions, P20, and P05.07 ( Halloun & Hestenes, 1996b). We used the current P05.07 version accessible from the PhysPort website. This version contains 33 items that measure students learning outcome before and after covering a certain content. VASS is not multiple-choice ^1^, items are formulated based on Halloun’s Contrasting Alternatives rating scale (Cars) and done in 40 minutes. Items are rated on a 5-point scale and each scale an ‘a’ revealing negative attitude and a ‘b’ statement revealing a positive attitude (see Figure 1). The first scale (a) >> (b) means mostly (a), rarely (b); the second scale (a) > (b) means more (a) than (b); the third scale (a) = (b) means equally (a) and (b); the fourth scale (b) > (a) means more (b) than (a); and the fifth scale (b) >> (a) means mostly (b), rarely (a).

To ensure content validity of the study instruments, the instrument was presented to four research experts before its adoption to ensure that it matched the problem under investigation as recommended by scores ( Gay, Mills & Airasian, 2012; Samsudin *et al.*, 2019). The experts were selected from Secondary Science and Mathematics Teachers (SESEMAT) association in Uganda, and from the department of science education in the University of Rwanda College of Education and Mbarara University of Science and Technology and were requested to indicate whether the items were relevant to the problem under investigation. After collecting their opinions on every item, the content validity ratio (C.V.R) for each item was calculated based on the formula by Lawshe (1975):

$$\text{C.V.R.}=\frac{n_{e-\frac{N}{2}}}{\frac{N}{2}}$$ (1)

where *n*_*e*_ is the number of panelists indicating “essential” and *N* is the total number of panelists.

In this study ( *N* = 4 and *n*_*e*_ = 3), when the C.V.R. values for all items were averaged, the items with the CVR bigger than 0.49 remained, and the Content Validity Index (C.V.I.) was obtained as 0.98. Comparing this value with the critical value of 0.99 ( Lawshe, 1975), the difference was considered good hence the instrument was considered valid.

Suppose a research instrument is to be considered reliable. In that case, it must prove that if it were to be used on a similar group of participants under the same conditions, it would still result in similar results ( Cohen *et al.,* 2007). Achievement of consistency gives the researcher assurance that the results obtained represent the achievement of the individual participants ( Fraenkel *et al.,* 2012). After the survey was accepted for adoption, a pilot study was administered to 42 randomly selected students among the study participants. The internal consistency of the study instrument was determined by computing the Cronbach’s Alpha which gave a 0.73 coefficient, and this value rendered it reliable according to Gliem and Gliem (2003). We have also employed and administered the mechanical waves concept survey ( Tongchai *et al.,* 2008), a valid and standard tool available at physport.com for triangulation purpose.

### Ethical considerations

After the approval of the proposal, an ethical clearance (Ref: 03/DRI-CE/067/EN/gi/2020) was obtained from research and innovation office, University of Rwanda, College of Education and thereafter an authorisation letter to conduct research in Uganda was given by the permanent secretary, Ministry of Education and Sports – Uganda. A week before the start of data collection, participants first signed written informed consent letters. Participants who were below 18 years were asked to consult their parents and in turn, their parents consented on their behalf. Each participant was given a code and was referred to only by that code. No monetary compensation was given to participants. Participants were free to withdraw from the study at any time without penalty and were also free not to answer any questions or respond to any research situations if they chose so.

### Data analysis

In VASS development, Halloun (1996) classified students’ views or attitude in three profiles; expert, transitional, and folk. We have based our analysis on these distinct categories. The most common way to score these surveys is to collapse students’ responses into two categories depending on whether they are the same as an expert physicist would give (called “percent expert like response” or “percent favorable response”) ( Madsen *et al.,* 2020). We used both MS Excel 2016 (Microsoft, 2016) (RRID:SCR_016137) and SPSS 23.0 (IBM Corp, 2015) (RRID:SCR_016479) to analyze data. We computed frequencies of students in each of the VASS categories (1-5) using “countif” function in Excel 2016. We then merged these five categories into three categories. Thus, we averaged the first two categories into folks-like attitude, last two categories into physics expert-like attitude, and the third (middle) category remained as it is and named transitional attitude. We averaged these frequencies across 33 VASS items to make displayable percentages along with the Solomon four groups. We then followed this procedure to compute frequencies across gender and school type. The “shift” in percent favorable responses is calculated by subtracting the pre-test class average percent favorable from the post-test class average percent favorable. This metric tells you how students’ expert-like or favorable beliefs about physics changed from the start to the end of their physics course ( Madsen *et al.,* 2020). To measure the correlation between students’ attitude and performance, we computed and compared the average scores of mechanical waves conceptual survey (MWCS) correct answers and average scores of VASS for each student. To measure the statistical significance between test groups and gender or school factors, we employed nonparametric tests in SPSS and computed Mann-Whitney U or Kruskal-Wallis tests where appropriate.

## Results

### Classification of students’ attitudes about learning physics before and after learning through PBL

**Figure 2.**
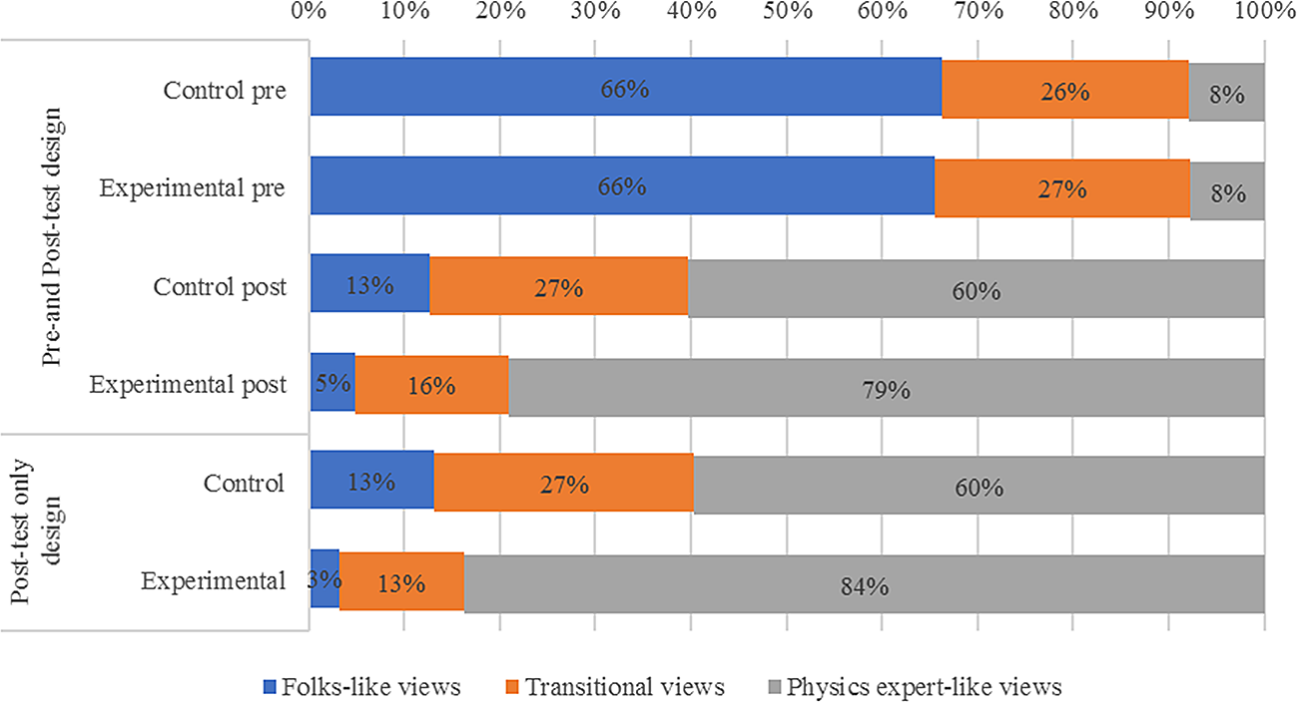
Classification of students’ attitude about learning physics before and after learning through problem-based learning.

Figure 2 displays the overall results of learning physics. By considering only the groups of students who sat for both pre-and post-tests, it is seen that most of the students (66%), within the experimental group, display high and similar attitude (folks-like attitude) before learning *via* PBL approach. However, after learning about mechanical waves, there was a great shift from attitude in both students in control and experimental groups. The difference between these two groups was 19% more students (79% of students in experimental and 60% of students in control group) changed their attitude towards physics expert-like due to PBL. It is also noted that in the second stream of students who did not sit for pre-test, only 3% of students taught using PBL, alongside 13% of those who were taught in traditional methods, had a folk-like attitude after completing the lessons.

**Figure 3.**
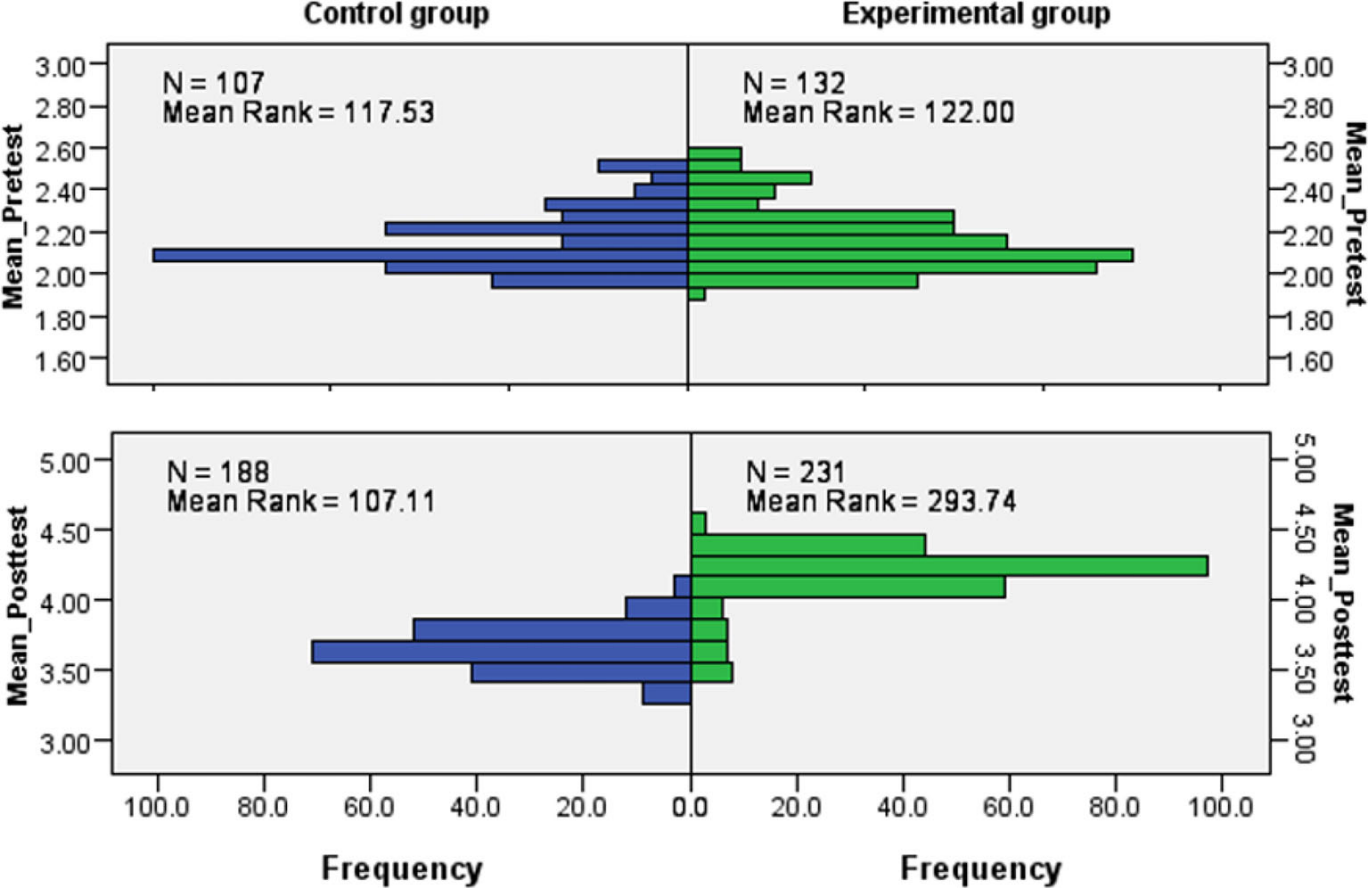
Independent-Samples Mann-Whitney U test across control and experimental groups. Note: y-axis shows average scores and x-axis shows number of students in %.

Table 1 presents the statistical significances of the groups discussed in Figure 3 above. The students’ attitude from either control or experimental groups was similar before learning mechanical waves. However, it diverged after learning, and a physics expert-like attitude was developed more in the experimental group. Therefore, we retained the hypothesis that assumed that the distribution of mean scores of all students was the same at the pre-test stage and rejected the hypothesis that assumed the distribution of mean scores of all students was the same at the post-test stage. Nevertheless, there was no statistically significant difference ( *p* > 0.05) between female and male students and between public and private schools both at pre- and post-test stages.

**Table 1.** Non-Parametric tests and hypothesis testing.

	Null hypothesis	Test used	N (df)	Significance
1	The distribution of the mean score of the Pre-test is the same across categories of “Experimental and Control groups”	Independent Samples Mann-Whitney U Test	239	0.618
2	The distribution of the mean score of Post-test is the same across categories of “Experimental and Control groups”	Independent Samples Mann-Whitney U Test	419	0.000 *
3	The distribution of the mean score of the Pre-test is the same across categories of “Solomon four-groups”	Independent Samples Kruskal-Wallis Test	239 (1)	0.618
4	The distribution of the mean score of the Post-test is the same across categories of “Solomon four-groups”	Independent Samples Kruskal-Wallis Test	419 (3)	0.000 *
5	The distribution of the mean score of the Pre-test is the same among female and male students	Independent Samples Mann-Whitney U Test	239	0.565
6	The distribution of the mean score of the Post-test is the same among female and male students	Independent Samples Mann-Whitney U Test	419	0.216
7	The distribution of the mean score of the Pre-test is the same among public and private schools	Independent Samples Mann-Whitney U Test	239	0.866
8	The distribution of the mean score of Post-test is the same among public and private schools	Independent Samples Mann-Whitney U Test	419	0.078

* Statistically significant difference at.05 significance level and rejecting the null hypothesis, N: Sample size, df: degrees of freedom.

Figure 2 displays the VASS mean scores of students in control and experimental groups at pre- and post-test stages. It can be visualized that students’ attitudes shifted to higher scores in the experimental group than the control group after learning mechanical waves (at the pre-test stage). Note that higher scores refer to a more “expert” like attitude.

**Figure 4.**
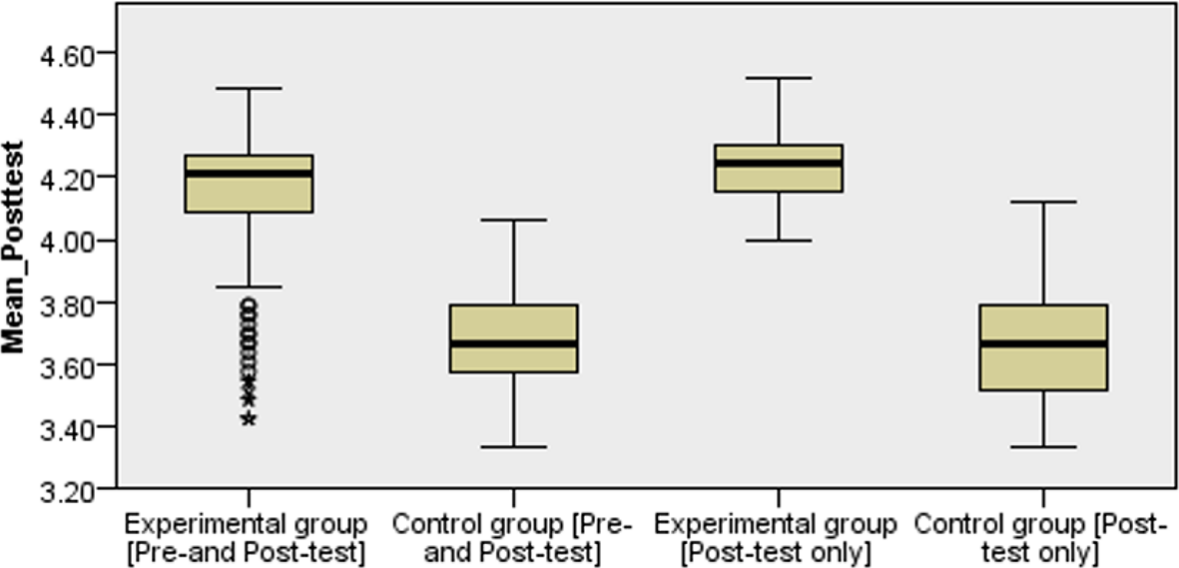
Independent-Samples Kruskal-Wallis test across Solomon four-group design.

Figure 4 displays box and whisker plots across Solomon’s four-group design. It is seen that the experimental group (those who learned with PBL) either performed both pre- and post-test or those who performed only post-test had similar high VASS average scores compared to their counterparts in the control group (those who learned with chalk and board).

We have investigated two factors (students’ gender and school type/ownership) that could influence students’ attitude toward learning physics when learned with or without PBL.

*Case 1.Gender difference*

**Figure 5.**
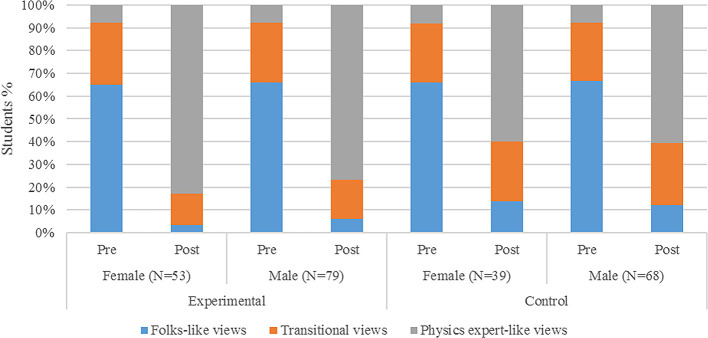
Classification of students’ attitude about learning physics before and after learning through PBL according to gender.

Since gender showed no statistical ( *p* > 0.05) effect (see Table 1), Figure 5 displays the classification of students’ attitude about learning physics before and after learning through PBL according to gender. Thus, both male and female students were able to shift from folks-like to physics expert-like attitude after learning mechanical waves. The sample for female students at both pre- and post-test was 53 while that of male was 79 in experimental group. Likewise, there wre 39 female and 68 male students in control group.

It can be seen that 65% female students had folk-like views in pre-test that shifted to 83% expert-like in post-test due to the PBL intervention. A similar shift in male students was observed from 66% to 77%, from folk to expert-like views. However, such big shift was not observed in a control group. For instance, the shift in expert-like views was from 8% to 60% and from 8% to 61% among females and males respectively.

*Case 2. Public versus private school*

**Figure 6.**
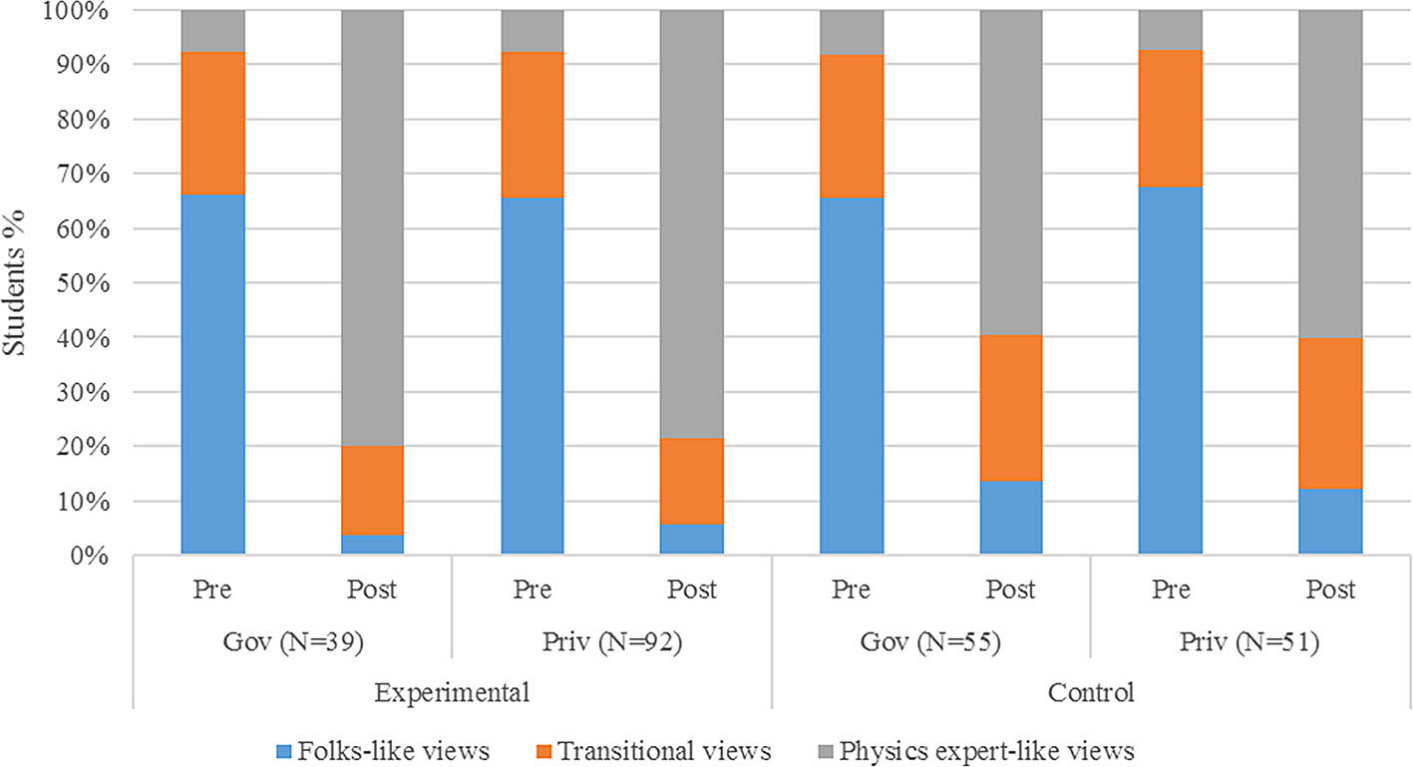
Classification of students’ attitude about learning physics before and after learning through PBL according to public or private school.

Since public and private schools did not show any statistical ( *p* > 0.05) effect (see Table 1), Figure 6 displays the classification of students’ attitude about learning physics before and after learning through PBL across the type of schools. Thus, students in public or private were able to shift from folks-like to physics expert-like attitude after learning mechanical waves. The sample for students from government school at both pre- and post-test was 39 while that of those from private was 92 in experimental group. Likewise, there were 55 students in government school and 51 students in private school.

It can be seen that 66% government students had folk-like views in pre-test, that shifted to 80% expert-like in post-test due to the PBL intervention. A similar shift in private students was observed from 66% to 78%, from folk to expert-like views. However, such big shift was not observed in a control group. For instance, there was only 60% students from government school and 60% students from private school shifted to expert-like views.

### Correlation between students’ attitude and performance

**Figure 7.**
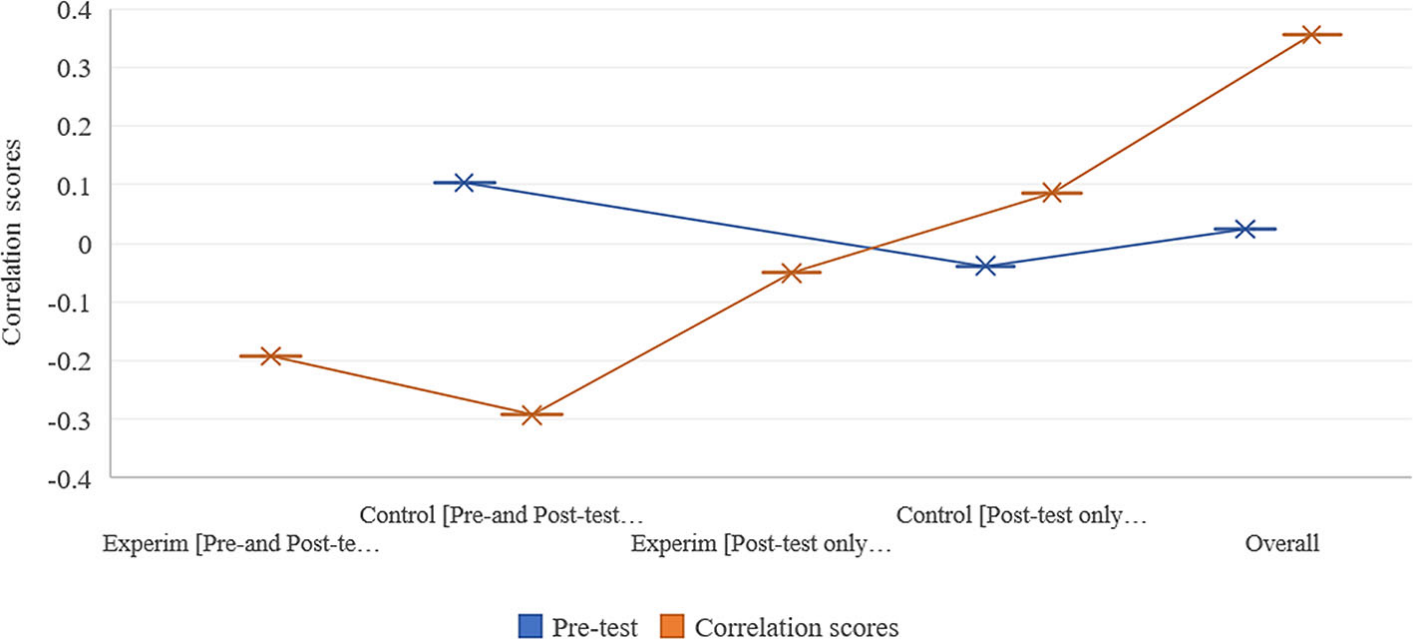
Correlation scores between MWCS and VASS across Solomon four-group design.

After measuring the effect of PBL on students’ attitude, we went further and correlated the scores from the MWCS and VASS scores. Figure 7 presents these results. Surprisingly, a positive attitude, such as physics expert-like, was found to not significantly correlate with students’ performance scores. A small correlation coefficient of 0.35 was found in overall students at the post-test stage. This was large compared with a very small correlation coefficient of 0.02 found in overall students at the pre- test stage. Note that a correlation between 0.00 and 0.49 is considered low, 0.50 and 0.69 is considered medium, while 0.70 and 1.00 is considered as high.

Specifically, a negative correlation (but very small) coefficient (-0.03) was found in a control group (in a post-test only design), while a positive correlation (but small) coefficient (0.10) was found in a control group (in a pre- and post-test design) at pre-test stage ( Table 2).

**Table 2.** Extended results from Figure 7.

	Pre-test	Post-test
Experim [Pre- and Post-test design]		-0.19
Control [Pre- and Post-test design]	0.10	-0.29
Experim [Post-test only design]		-0.05
Control [Post-test only design]	-0.04	0.09
Overall	0.02	0.36

Likewise, a series of negative correlation coefficients (such as -0.19 found in Experim group of pre- and post-test design) were found at the post-test stage with only a positive correlation (but a small) coefficient (.08) in a control group that performed only post-test.

## Discussion

Attitude is one of the best behavior characteristics that can show the orientation of the course during teaching and learning. We have investigated the effect of PBL on improving the attitude in learning physics among Ugandan students. We found that PBL raises students toward learning physics more than traditional methods do (N = 419, *p* > 0.05). Students shifted from folks-like attitudes before learning mechanical waves to physics expert-like after learning mechanical waves. These results our study tend to agree with those of Laforce and Noble (2017) who found that significantly enhanced students’ motivation and beliefs about STEM careers. In a similar way, PBL method of teaching was found to have a stronger positive impact in the general perception of Pharmacy students towards the study of anatomy as compared to the traditional methods in the study by Azu and Osinubi (2011). However, Sahin (2010) did not find a significant difference in students’ beliefs about learning physics between those instructed under PBL and those instructed under traditional lecture method. The expert-like attitude are attitudes we believe are shared by scientists and educators at large ( Halloun, 1996). This author argued that students with an expert profile are chiefly scientific realists and critical learners. Students with a folk profile are primarily naive realists and passive learners ( Madsen *et al.,* 2020). Students with transitional profiles hold mixtures of this attitude.

In a study done in Uganda, Kwarikunda *et al.* (2021) investigated motivation pertains of high school students toward learning physics and found that low-quantity and grade-introjected motivated students mostly used surface learning strategies whilst the high-quantity and primarily intrinsically motivated students used deep learning strategies. This is related to our findings in a way that the PBL strategy has raised motivation of students to learn physics. The shift towards physicist-like attitude were also realized during examining the attitude of physics experts, physics educationists in the incorporation of history and philosophy of science-based materials in physics instruction ( Galili & Hazan, 2001). The authors of that study realized that such knowledge could guide those who devote their efforts to constructing and implementing learning materials in science education. These findings indicate that not only students but also teachers may experience such folks-like beliefs as pointed out by Zhukova (2017) in his study findings which suggested that some teachers especially the beginners tend to hold traditional beliefs or feel incompetent in relation to using learner-centered methods although they tend to hind their fears due to student expectations among other factors.

With reference to Figure 2, although students’ views in the post test were more of expert-like especially with the experimental group, some students still possessed folk-like and transitional views despite being instructed with PBL method. I this regard, the VASS study ( Halloun, 1996), also found that college physics students have attitude about physics that often diverge from physicists’ attitude, with the majority of students evincing a transitional profile. Such results may persist due to the fact that some students may naturally have low interest rate, expectation or success towards physics as a subject according to Boyuk (2011).

In our study, gender and type of school did not show any effect of changing attitude or supporting PBL to change students’ attitude toward physics expert-like thinking. Thus, attitude changes with instruction rather than on gender or school factors. Other studies such as Argaw *et al.* (2017) did not find any gender difference in motivation to learn physics across groups, thus, there was no domination of gender existed in the results obtained in both the experimental and comparison groups. However, a comparison study done in Turkey about PBL and traditional lecture students’ expectations in an introductory physics classroom showed that there was a sense that the attitude towards physics may change with gender ( Sahin & Yorek, 2009). Likewise, Mbonyiryivuze *et al.* (2021) found a statistically relevant gender gap in favor of female students about the use of learning physics in helping to understand situations in students’ everyday life.

One of the findings in our study that builds the gap in the current literature is that we found a non-significant difference between public and private schools in attitude when high school students experience PBL instruction. Existing literature ( Maison *et al.,* 2021) has shown that public junior high school students have slightly more positive attitudes than private students when learning science in general. Likewise, in relation to school levels, Mukagihana *et al.* (2021) found an increased motivation of learning biology at a public university than at a private university when animation and lab instructions are administered. We have also measured the correlation between students’ attitude and performance. Sometimes students’ attitude may significantly affect what they learn in science courses ( Halloun & Hestenes, 1996a); however, our results show no such correlation (r < 0.50). This is surprising because everyone would predict the positive correction with performance after seeing the increase in students’ attitude as our study revealed. Note that more results about students’ achievement are reported in our study ( Kanyesigye *et al.,* 2022a). Contrariwise, other studies have seen a significant correlation between attitude and scores. For instance, Halloun and Hestenes (1996a) study, student profiles correlated significantly with physics achievement (r > 0.441). Students with an expert profile were the most likely to earn an “A” in their physics courses, while those with a folk profile are the most likely to do poorly or fail in these courses ( Halloun, 1996).

## Conclusions

In this study, we aimed to investigate whether problem-based learning instruction causes a statistically significant effect in students’ attitudes towards physics, whether there is a statistically significant difference by gender in students’ attitude towards physics, whether there is a statistically significant difference by school type in students’ attitude towards physics, and whether students’ attitude correlates with their performance scores. We found that students’ attitudes increase drastically after learning mechanical waves, developing physics expert-like profiles. There was no significant effect on the attitudes of students based on gender and school type. However, the correlation between positive attitudes did not highly correlate with their performance. We recommend teachers keep using problem-based learning techniques to develop students who possess a scientific profile. When attitude shifts are negative, teachers can change their teaching techniques to help develop students’ beliefs to be more expert-like. When differences in attitudes and beliefs scores are found in demographics, teachers should look for ways to support students in a more equitable manner.

## Data availability

We have previously published a data article in Data in Brief ( Kanyesigye *et al.,* 2022b) and full description of the data can be found in this study.

### Underlying data

Mendeley Data: Data for measuring impact of problem-based learning during learning mechanical waves: MWCS, VASS, RTOP. https://doi.org/10.17632/rdtcgstmps.3 ( Kanyesigye *et al.,* 2022c)

This project contains the following underlying data:

- Ugandan Secondary Form 6 Students Views About Sciences Survey [Feb-Apr 2021].xlsx
- Ugandan Secondary Form 6 Responses on Mechanical Wave Conceptual Survey [Feb-Apr 2021].xlsx

### Extended data

Mendeley Data: Teacher Training in Implementing Problem-Based Learning. https://doi.org/10.17632/b28d3p7kf8.1 ( Kanyesigye, 2022)

This project contains the following extended data.

- Training of Teachers in Problem-Based Learning.pptx

Mendeley Data: Data for measuring impact of problem-based learning during learning mechanical waves: MWCS, VASS, RTOP. https://doi.org/10.17632/rdtcgstmps.3 ( Kanyesigye *et al.,* 2022c)

This project contains the following extended data.

- Reformed teaching observation classroom practices in Ugandan Secondary Form 6 [Feb-Apr 2021].xlsx

Data are available under the terms of the Creative Commons Attribution 4.0 International license (CC-BY 4.0).

## Studies related to this current study

The current study is a portion of the first author doctoral research project. There are other studies by the authors related to this one that may overlap the methods or data. These are:

Published:

Kanyesigye, S. T., & Kemeza, I. (2021). Effect Of Problem-Based Learning Instruction On Secondary School Physics Students In Understanding Of Electromagnetic Waves. *Voice of Research*, *10*(1), 1–17. http://www.voiceofresearch.org/Doc/Jun-2021/Jun-2021_1.pdf

Kanyesigye, S. T., Uwamahoro, J., & Kemeza, I. (2022a). Difficulties in understanding mechanical waves: Remediated by problem-based instruction. *Physical Review Physics Education Research.* https://journals.aps.org/prper/accepted/99074L91A641480370857b82c918eea0b009ef16e

Kanyesigye, T. S., Uwamahoro, J., & Kemeza, I. (2022b). Data collected to measure the impact of problem-based learning and document physics classroom practices among Ugandan secondary schools. *Data in Brief*, *44*(108534 Contents), 1–9. https://doi.org/10.1016/j.dib.2022.108534

Accepted manuscript:

Kanyesigye, S. T., Uwamahoro, J., & Kemeza, I. (2022). The effect of Professional Training on In-service Secondary School Physics Teachers’ Motivation to Use Problem-Based Learning. *International Journal of Learning, Teaching and Educational Research (IJLTER)*

Under review:

Kanyesigye, S. T., Uwamahoro, J., & Kemeza, I. (2022). The Impact of Problem-Based Learning on Students’ Achievement in Mechanical Waves in Secondary Schools. *Research in Science Education (RISE).*

Manuscript in preparation:

Kanyesigye, S. T., Uwamahoro, J., & Kemeza, I. (2022). Ugandan physics classroom observation practices documented using reformed teaching observation protocol.

## Footnotes

^1^
The overall score on the attitudes and beliefs questionnaire is a measure of how well students agree with physicists, while the overall score on the multiple-choice conceptual test measures how well students understand physics content ( Madsen *et al., *2020, p.91).
