# Supplementary material for: Effect of problem-based learning on students’ attitude towards learning physics: a cohort study
Source: F1000Res. 2022 Nov 1;11:1240. [Version 1] doi: 10.12688/f1000research.125085.1 (PMC10721964; doi:10.12688/f1000research.125085.1)
Supplement: Peer review history

# Reviewer report: Reviewer response for version 1

**DOI:** 10.5256/f1000research.137353.r191679

**Published:** 2023-12-14

**Authors:** KINGSLEY T. ONAH

**Affiliations:** 1 Enugu State University of Science and Technology, Enugu, Enugu, Nigeria

Abstract: state the problem of the study before purpose as done.

 introduction: Present as thus: Physics and its relevance

 attitude level of the students; if there is any poor attitude amongst students, link it with any kind of instructions that had led to these abysmal attainment in the students attitude.

 then, introduce problem based learning strategy.

 gender if necessary.

 substantiate the choice of the study area. what problem existed in the area as regard the students level of attitude that occasioned the study.

 use analytical tool of mean and standard deviation to answer the research questions and ANCOVA to test the hypotheses.

Is the work clearly and accurately presented and does it cite the current literature?

Partly

If applicable, is the statistical analysis and its interpretation appropriate?

No

Are all the source data underlying the results available to ensure full reproducibility?

Yes

Is the study design appropriate and is the work technically sound?

Partly

Are the conclusions drawn adequately supported by the results?

Yes

Are sufficient details of methods and analysis provided to allow replication by others?

No

Reviewer Expertise:

Science Education/Physics, students achievement in physics, self-efficacy, interest, motivation, procrastination, teachers support, innovative teaching approach.

I confirm that I have read this submission and believe that I have an appropriate level of expertise to confirm that it is of an acceptable scientific standard.

# Reviewer report: Reviewer response for version 1

**DOI:** 10.5256/f1000research.137353.r210936

**Published:** 2023-11-29

**Authors:** Hasan Şahin Kızılcık

**Affiliations:** 1 Gazi University, Ankara, Ankara, Turkey

The paper discusses how problem-based learning (PBL) education affected students' attitudes toward physics, as well as how those attitudes and physics performance are related. 

 Abstract:

 You should clearly write which method you mean by traditional instruction.

 Literature review:

 Low relevance to the main problem. I did not understand the link between this study and STEM. STEM is an interdisciplinary approach, but only physics was analysed here. In addition, while there is rich literature on PBL and attitudes, these are barely mentioned. A focused review of the literature would increase the comprehensibility and impact of the study.

 Objectives:

 In the study, comparisons were made according to school type and gender. However, there is no objective for these.

 Sampling:

 The sample contains the phrase "13th grade (aged between 16 and 21)". Is this not a very wide age range for a grade level? Is there a specific reason for this? There are significant differences between the cognitive and developmental characteristics of a 21-year-old student and a 16-year-old student.

 Implementation of the intervention:

 During the intervention, I understood that PBL sessions about waves were carried out with the students. However, I could not find any information about what kind of problem they were trying to solve. The content of the problem, its level and what exactly was expected from the students were not explained.

 Data collection methods:

 The structure of the survey is presented quite complex. It could have been presented more simply. Validity and reliability studies are adequately explained. However, I did not see information about the construct validity of the survey. Is the survey a single factor?

 Data analysis:

 The first and third categories are worth two points each and the middle category is worth one point. What is the reason for not keeping the category range equal when dividing the pentad structure into three categories?

 Why did you use nonparametric methods? Did your data not fulfill the prerequisites of parametric methods?

 Results:

 The presentation of the results is very clear.

 Discussion:

 The discussion section is organised to cover all aspects of the study. However, the literature used in the discussion and the educational level of the study are different. In the discussion, studies on university students were generally mentioned. However, the study was conducted with high school students. Studies at similar educational levels should also be included.

Is the work clearly and accurately presented and does it cite the current literature?

Partly

If applicable, is the statistical analysis and its interpretation appropriate?

Yes

Are all the source data underlying the results available to ensure full reproducibility?

Yes

Is the study design appropriate and is the work technically sound?

Partly

Are the conclusions drawn adequately supported by the results?

Yes

Are sufficient details of methods and analysis provided to allow replication by others?

Partly

Reviewer Expertise:

Physics education, Problem-based laearning

I confirm that I have read this submission and believe that I have an appropriate level of expertise to confirm that it is of an acceptable scientific standard, however I have significant reservations, as outlined above.

# Reviewer report: Reviewer response for version 1

**DOI:** 10.5256/f1000research.137353.r165348

**Published:** 2023-03-22

**Authors:** Edy Shahali

**Affiliations:** 1 Faculty of Education, University of Malaya, Kuala Lumpur, Malaysia

The manuscript describes the effect of problem-based learning (PBL) instruction on students’ attitudes towards physics, and how students’ attitudes towards physics correlate with students' performance in physics.  

 Literature review **:** Literature related to how attitudes effect student performance should be described. The writing is found to tend to focus on how attitudes effect interest towards science/physics rather that how attitudes effect performance (which is the second objective of this study).

 The need or justification for the importance of conducting this study needs to be highlighted by presenting the relevant gaps in past studies.

 ​

 Researchers were found to use the words "STEM", "science" and "physics" interchangeably throughout the writing. It is necessary to examine the appropriateness of its use because it may give different interpretations to the reader. It is recommended that the writing be focused on learning physics which is the context of this study.

 Objective: There are no objectives related to differences in attitudes based on gender and type of school stated in this section. However, in the Results section, there are findings related to differences in attitudes towards gender and type of school were reported. Therefore, it is necessary to revise the writing of the objective to be in line with the reported research findings.

 Methodology: The appropriateness of this sentence needs to be reviewed. " *So, the author took up the topic of waves for the PhD project *".What does this statement have to do with this study need to be checked.

 Implementation: The number of physics teachers reported in the Implementation section (10) does not match the number of physics teachers trained (30) as stated in the Methodology section. It is necessary to explain why there is such a difference.

 The researchers stated that " *10 physics teachers who handled the experimentation group first trained students about PBL strategies before starting the experiment*."  - How the students were trained needs to be explained.

 The researchers stated that " *each member was expected to make individual study*" -  What is meant by "individual study' and how it is carried out need to be explained.

 Result: The researchers were found to report an analysis related to students' attitudes towards physics (when learned with or without PBL) based on gender and school type/ownership. However, objective of the study does not state this. Therefore, it is necessary to revise the writing of the objective to be in line with the reported research findings.

 Discussion: Writing in the discussion section needs to be re-examined and needs to be written more comprehensively by presenting possible reasons for the reported findings. Researchers are found to only report the findings of past studies that are parallel and not parallel to their research findings. No arguments are presented to explain the findings. 

 The researchers was also found to be comparing the findings of their study with the findings of previous studies that involved factors such as *belief*, *motivation*, and *perceptions*, rather than comparing past studies related to *attitudes*. Therefore, it is necessary to check its appropriateness.

 The researchers stated that " *This is related to our findings in a way that the PBL strategy has raised motivation of students to learn physics*". However, this study is related to the attitude towards physics and no data related to the level of motivation was obtained in this study.

 The researchers stated that " *our results show no such correlation between attitudes and achievement*". However, no possible reason to explain this was discussed. 

 Conclusion - There are some statements that are not consistent with the findings of the study and need to be revised. Among them, the researchers were found to draw conclusions about *beliefs*, whereas this study only identified the level of students' attitudes towards physics.

 Thank you.

Is the work clearly and accurately presented and does it cite the current literature?

Partly

If applicable, is the statistical analysis and its interpretation appropriate?

Partly

Are all the source data underlying the results available to ensure full reproducibility?

Yes

Is the study design appropriate and is the work technically sound?

Partly

Are the conclusions drawn adequately supported by the results?

Partly

Are sufficient details of methods and analysis provided to allow replication by others?

Partly

Reviewer Expertise:

Science education, STEM education, teacher professional development

I confirm that I have read this submission and believe that I have an appropriate level of expertise to confirm that it is of an acceptable scientific standard, however I have significant reservations, as outlined above.
